# *catena*-Poly[[silver(I)-[μ-4-(2-pyrid­yl)­pyrimidine-2-sulfonato]] monohydrate]

**DOI:** 10.1107/S1600536810012596

**Published:** 2010-04-10

**Authors:** Hai-Bin Zhu

**Affiliations:** a School of Chemistry and Chemical Engineering, Southeast University, Nanjing, People’s Republic of China

## Abstract

In the title compound, {[Ag(C_9_H_6_N_3_O_3_S)]·H_2_O}_*n*_, the Ag^I^ atom is coordinated by three N atoms and two sulfonate O atoms from two different 4-(2-pyrid­yl)pyrimidine-2-sulfonate ligands. The ligand bridges two Ag^I^ atoms, forming a polymeric zigzag chain propagating parallel to [001]. The uncoordinated water mol­ecule is involved in hydrogen bonds with sulfonate O atoms.

## Related literature

For our previous work with the 4-(2-pyrid­yl)pyrimidine-2-sulfonate ligand, see: Zhu *et al.* (2007 ▶).
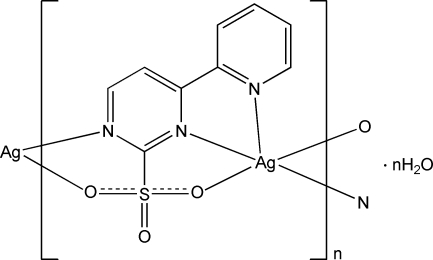


## Experimental

### 

#### Crystal data

[Ag(C_9_H_6_N_3_O_3_S)]·H_2_O
*M* *_r_* = 362.12
Monoclinic, 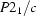

*a* = 6.9020 (3) Å
*b* = 13.6228 (6) Å
*c* = 12.1337 (5) Å
β = 99.975 (2)°
*V* = 1123.62 (8) Å^3^
*Z* = 4
Mo *K*α radiation
μ = 1.99 mm^−1^
*T* = 298 K
0.18 × 0.15 × 0.12 mm

#### Data collection

Bruker APEXII CCD diffractometer
Absorption correction: multi-scan (*SADABS*; Bruker, 2001 ▶) *T*_min_ = 0.706, *T*_max_ = 0.788
6630 measured reflections
2533 independent reflections
2160 reflections with *I* > 2σ(*I*)
*R*_int_ = 0.015

#### Refinement

*R*[*F* ^2^ > 2σ(*F* ^2^)] = 0.024
*wR*(*F* ^2^) = 0.071
*S* = 1.02
2533 reflections
163 parameters
H-atom parameters constrained
Δρ_max_ = 0.51 e Å^−3^
Δρ_min_ = −0.53 e Å^−3^

### 

Data collection: *APEX2* (Bruker, 2007 ▶); cell refinement: *SAINT-Plus* (Bruker, 2007 ▶); data reduction: *SAINT-Plus*; program(s) used to solve structure: *SHELXS97* (Sheldrick, 2008 ▶); program(s) used to refine structure: *SHELXL97* (Sheldrick, 2008 ▶); molecular graphics: *SHELXTL* (Sheldrick, 2008 ▶); software used to prepare material for publication: *SHELXTL*.

## Supplementary Material

Crystal structure: contains datablocks I, global. DOI: 10.1107/S1600536810012596/hy2295sup1.cif

Structure factors: contains datablocks I. DOI: 10.1107/S1600536810012596/hy2295Isup2.hkl

Additional supplementary materials:  crystallographic information; 3D view; checkCIF report

## Figures and Tables

**Table 1:** Selected bond lengths (Å)

Ag1—N1^i^	2.279 (2)
Ag1—N2	2.393 (2)
Ag1—N3	2.337 (2)
Ag1—O1^i^	2.668 (2)
Ag1—O3	2.693 (2)

Symmetry code: (i) 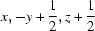.

**Table 2:** Hydrogen-bond geometry (Å, °)

*D*—H⋯*A*	*D*—H	H⋯*A*	*D*⋯*A*	*D*—H⋯*A*
O4—H2⋯O3	0.85	1.95	2.793 (4)	173
O4—H1⋯O2^ii^	0.85	2.07	2.913 (4)	173

Symmetry code: (ii) 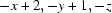.

**Fig. 1.**
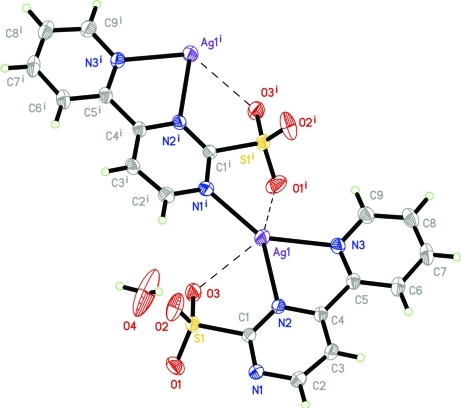
The coordination environment around AgI in the title complex. Displacement ellipsoids are drawn at the 30% probability level. The Ag···O bonds are shown as dashed lines. [Symmetry code: (i) x, 1/2-y, 1/2+z.]

## supplementary crystallographic information

### Comment

In our previous work, we have reported several divalent transition metal coordination compounds with 4-(2-pyridyl)pyrimidine-2-sulfonate (*L*) ligand (Zhu *et al.*, 2007). Herein, we present a new silver(I) coordination polymer with *L*.

The title compound has a polymeric zigzag chain structure, where the Ag^I^ atom is penta-coordinated by three N atoms and two sulfonate O atoms from two *L* ligands (Fig. 1). The Ag—N bond lengths vary between 2.279 (2) and 2.393 (2) Å, and the Ag—O distances are in the range of 2.668 (2) and 2.693 (2)Å (Table 1). The uncoordinated water molecule is involved in hydrogen bonds with sulfonate O atoms (Table 2).

### Experimental

A colorless solution of AgNO_3_ (0.017 g, 0.1 mmol) in CH_3_CN (5 ml) was carefully layered onto a solution of 4-(2-pyridyl)pyrimidine-2-sulfonic acid (0.026 g, 1 mmol) in H_2_O (5 ml). Diffusion between the two phases over a period of 5 d produced light-yellow crystals (yield: 0.026 g, 72% based on silver nitrate).

### Refinement

H atoms bounded to C atoms were positioned geometrically and allowed to ride on their parent atoms, with C—H =0.93 Å and with *U*_iso_(H) = 1.2*U*_eq_(C). The positions of the water H atoms were found from a difference Fourier map and refined as riding with O—H = 0.85 Å and *U*_iso_(H) = 1.2*U*_eq_(O).

### Crystal data

**Table:**

[Ag(C_9_H_6_N_3_O_3_S)]·H_2_O	*F*(000) = 712
*M**_r_* = 362.12	*D*_x_ = 2.147 Mg m^−^^3^
Monoclinic, *P*2_1_/*c*	Mo *K*α radiation, λ = 0.71073 Å
Hall symbol: -P 2ybc	Cell parameters from 2533 reflections
*a* = 6.9020 (3) Å	θ = 2.3–27.5°
*b* = 13.6228 (6) Å	µ = 1.99 mm^−^^1^
*c* = 12.1337 (5) Å	*T* = 298 K
β = 99.975 (2)°	Block, colorless
*V* = 1123.62 (8) Å^3^	0.18 × 0.15 × 0.12 mm
*Z* = 4	

### Data collection

**Table:**

Bruker APEXII CCD diffractometer	2533 independent reflections
Radiation source: fine-focus sealed tube	2160 reflections with *I* > 2σ(*I*)
graphite	*R*_int_ = 0.015
φ and ω scans	θ_max_ = 27.5°, θ_min_ = 2.3°
Absorption correction: multi-scan (*SADABS*; Bruker, 2001)	*h* = −8→8
*T*_min_ = 0.706, *T*_max_ = 0.788	*k* = −17→15
6630 measured reflections	*l* = −15→13

### Refinement

**Table:**

Refinement on *F*^2^	Primary atom site location: structure-invariant direct methods
Least-squares matrix: full	Secondary atom site location: difference Fourier map
*R*[*F*^2^ > 2σ(*F*^2^)] = 0.024	Hydrogen site location: inferred from neighbouring sites
*wR*(*F*^2^) = 0.071	H-atom parameters constrained
*S* = 1.02	*w* = 1/[σ^2^(*F*_o_^2^) + (0.0401*P*)^2^ + 0.5886*P*] where *P* = (*F*_o_^2^ + 2*F*_c_^2^)/3
2533 reflections	(Δ/σ)_max_ < 0.001
163 parameters	Δρ_max_ = 0.51 e Å^−^^3^
0 restraints	Δρ_min_ = −0.53 e Å^−^^3^

### Fractional atomic coordinates and isotropic or equivalent isotropic displacement parameters (Å^2^)

**Table:**

	*x*	*y*	*z*	*U*_iso_*/*U*_eq_	
Ag1	0.81252 (4)	0.174975 (15)	0.078449 (17)	0.04992 (10)	
N2	0.7873 (3)	0.14417 (14)	−0.11767 (17)	0.0335 (4)	
N3	0.7593 (3)	0.00808 (15)	0.04023 (18)	0.0389 (4)	
C3	0.7075 (4)	0.03190 (19)	−0.2672 (2)	0.0420 (6)	
H3A	0.6753	−0.0311	−0.2936	0.050*	
C4	0.7418 (3)	0.05178 (16)	−0.1536 (2)	0.0333 (5)	
C6	0.6871 (4)	−0.1210 (2)	−0.0934 (3)	0.0480 (6)	
H6A	0.6683	−0.1416	−0.1675	0.058*	
C9	0.7459 (4)	−0.0571 (2)	0.1213 (3)	0.0477 (6)	
H9A	0.7662	−0.0355	0.1950	0.057*	
C8	0.7034 (4)	−0.1546 (2)	0.1002 (3)	0.0527 (7)	
H8A	0.6952	−0.1977	0.1586	0.063*	
C7	0.6733 (4)	−0.1873 (2)	−0.0083 (3)	0.0544 (8)	
H7A	0.6440	−0.2529	−0.0246	0.065*	
C5	0.7293 (3)	−0.02331 (18)	−0.0662 (2)	0.0359 (5)	
S1	0.85817 (10)	0.33521 (4)	−0.14563 (5)	0.04032 (15)	
N1	0.7693 (3)	0.19927 (16)	−0.30431 (18)	0.0410 (5)	
C1	0.7996 (3)	0.21175 (17)	−0.1937 (2)	0.0338 (5)	
O2	0.6717 (4)	0.38366 (17)	−0.1628 (2)	0.0786 (8)	
C2	0.7221 (4)	0.1074 (2)	−0.3396 (2)	0.0463 (6)	
H2B	0.6986	0.0947	−0.4160	0.056*	
O1	0.9932 (4)	0.36957 (18)	−0.21511 (19)	0.0694 (7)	
O3	0.9460 (3)	0.32665 (13)	−0.02960 (17)	0.0521 (5)	
O4	1.2896 (6)	0.4143 (3)	0.0834 (5)	0.189 (3)	
H2	1.1797	0.3920	0.0504	0.226*	
H1	1.3116	0.4722	0.1086	0.226*	

### Atomic displacement parameters (Å^2^)

**Table:**

	*U*^11^	*U*^22^	*U*^33^	*U*^12^	*U*^13^	*U*^23^
Ag1	0.07851 (18)	0.03703 (13)	0.03468 (13)	0.00054 (9)	0.01113 (10)	−0.00254 (7)
N2	0.0349 (10)	0.0297 (9)	0.0368 (11)	−0.0010 (8)	0.0082 (8)	−0.0028 (8)
N3	0.0411 (11)	0.0335 (10)	0.0430 (11)	0.0032 (9)	0.0096 (9)	0.0039 (9)
C3	0.0486 (14)	0.0329 (12)	0.0455 (14)	−0.0056 (11)	0.0107 (11)	−0.0092 (10)
C4	0.0272 (11)	0.0300 (11)	0.0432 (13)	0.0012 (8)	0.0076 (9)	−0.0016 (9)
C6	0.0498 (15)	0.0319 (13)	0.0616 (18)	−0.0013 (11)	0.0077 (13)	−0.0046 (11)
C9	0.0497 (15)	0.0428 (14)	0.0526 (16)	0.0047 (12)	0.0144 (12)	0.0088 (12)
C8	0.0436 (15)	0.0419 (14)	0.074 (2)	0.0054 (11)	0.0144 (14)	0.0200 (14)
C7	0.0494 (16)	0.0296 (13)	0.083 (2)	0.0000 (11)	0.0094 (15)	0.0069 (13)
C5	0.0283 (11)	0.0305 (11)	0.0496 (14)	0.0021 (9)	0.0091 (10)	0.0000 (10)
S1	0.0537 (4)	0.0295 (3)	0.0366 (3)	−0.0036 (3)	0.0045 (3)	−0.0018 (2)
N1	0.0530 (13)	0.0370 (10)	0.0342 (11)	−0.0035 (9)	0.0110 (9)	−0.0035 (8)
C1	0.0352 (11)	0.0318 (11)	0.0354 (12)	−0.0005 (9)	0.0089 (9)	−0.0030 (9)
O2	0.0733 (15)	0.0478 (13)	0.105 (2)	0.0202 (11)	−0.0121 (14)	−0.0218 (13)
C2	0.0590 (16)	0.0432 (14)	0.0368 (13)	−0.0060 (12)	0.0087 (11)	−0.0094 (11)
O1	0.0992 (17)	0.0608 (14)	0.0516 (12)	−0.0412 (13)	0.0224 (12)	−0.0040 (10)
O3	0.0732 (13)	0.0410 (11)	0.0394 (11)	−0.0113 (9)	0.0021 (9)	−0.0044 (8)
O4	0.113 (3)	0.114 (3)	0.304 (7)	0.030 (2)	−0.063 (4)	−0.126 (4)

### Geometric parameters (Å, °)

**Table:**

Ag1—N1^i^	2.279 (2)	C9—C8	1.374 (4)
Ag1—N2	2.393 (2)	C9—H9A	0.9300
Ag1—N3	2.337 (2)	C8—C7	1.370 (5)
Ag1—O1^i^	2.668 (2)	C8—H8A	0.9300
Ag1—O3	2.693 (2)	C7—H7A	0.9300
N2—C1	1.317 (3)	S1—O2	1.429 (2)
N2—C4	1.351 (3)	S1—O3	1.438 (2)
N3—C9	1.340 (3)	S1—O1	1.439 (2)
N3—C5	1.342 (3)	S1—C1	1.803 (2)
C3—C2	1.368 (4)	N1—C1	1.333 (3)
C3—C4	1.384 (3)	N1—C2	1.344 (3)
C3—H3A	0.9300	N1—Ag1^ii^	2.279 (2)
C4—C5	1.487 (3)	C2—H2B	0.9300
C6—C7	1.388 (4)	O4—H2	0.8500
C6—C5	1.389 (4)	O4—H1	0.8500
C6—H6A	0.9300		
			
N1^i^—Ag1—N3	145.30 (7)	C8—C9—H9A	118.5
N1^i^—Ag1—N2	139.26 (7)	C7—C8—C9	119.0 (3)
N3—Ag1—N2	69.52 (7)	C7—C8—H8A	120.5
O1^i^—Ag1—N2	146.68 (7)	C9—C8—H8A	120.5
O1^i^—Ag1—N3	89.82 (7)	C8—C7—C6	119.0 (3)
O1^i^—Ag1—N1^i^	71.05 (8)	C8—C7—H7A	120.5
O3—Ag1—N1^i^	79.63 (7)	C6—C7—H7A	120.5
O3—Ag1—N2	67.83 (7)	N3—C5—C6	121.6 (2)
O3—Ag1—N3	134.83 (6)	N3—C5—C4	116.7 (2)
C1—N2—C4	117.7 (2)	C6—C5—C4	121.7 (2)
C1—N2—Ag1	124.89 (16)	O2—S1—O3	113.36 (16)
C4—N2—Ag1	117.21 (15)	O2—S1—O1	115.00 (18)
C9—N3—C5	118.4 (2)	O3—S1—O1	113.20 (14)
C9—N3—Ag1	121.87 (19)	O2—S1—C1	103.76 (13)
C5—N3—Ag1	119.59 (16)	O3—S1—C1	105.92 (11)
C2—C3—C4	118.3 (2)	O1—S1—C1	104.21 (12)
C2—C3—H3A	120.9	C1—N1—C2	115.2 (2)
C4—C3—H3A	120.9	C1—N1—Ag1^ii^	121.18 (17)
N2—C4—C3	119.6 (2)	C2—N1—Ag1^ii^	123.49 (17)
N2—C4—C5	116.7 (2)	N2—C1—N1	126.8 (2)
C3—C4—C5	123.7 (2)	N2—C1—S1	117.60 (18)
C7—C6—C5	119.0 (3)	N1—C1—S1	115.62 (18)
C7—C6—H6A	120.5	N1—C2—C3	122.4 (2)
C5—C6—H6A	120.5	N1—C2—H2B	118.8
N3—C9—C8	122.9 (3)	C3—C2—H2B	118.8
N3—C9—H9A	118.5	H2—O4—H1	126.2

Symmetry codes: (i) *x*, −*y*+1/2, *z*+1/2; (ii) *x*, −*y*+1/2, *z*−1/2.

### Hydrogen-bond geometry (Å, °)

**Table:**

*D*—H···*A*	*D*—H	H···*A*	*D*···*A*	*D*—H···*A*
O4—H2···O3	0.85	1.95	2.793 (4)	173
O4—H1···O2^iii^	0.85	2.07	2.913 (4)	173

Symmetry codes: (iii) −*x*+2, −*y*+1, −*z*.
